# Paired reentries maintain ventricular tachycardia: a topological analysis of arrhythmic mechanisms using the index theorem

**DOI:** 10.3389/fnetp.2025.1638085

**Published:** 2025-10-17

**Authors:** Robin Van Den Abeele, Sebastiaan Lootens, Bjorn Verstraeten, Arthur Santos Bezerra, Arstanbek Okenov, Timur Nezlobinskii, Viktor Van Nieuwenhuize, Sander Hendrickx, Nele Vandersickel

**Affiliations:** Department of Physics and Astronomy, Ghent University, Ghent, Belgium

**Keywords:** atrial tachycardia, topological charge, counter-clockwise, clockwise, tachycardia cycle length, local activation time, critical boundary, non-critical boundary

## Abstract

**Introduction:**

In previous research on reentrant atrial tachycardia (AT), the index theorem has proven instrumental in uncovering consistent paired counter-rotating anatomical reentry (either complete or near-complete), driving the arrhythmia rotating around critical boundaries (CB). Furthermore, interconnecting each CB-pair with an ablation line has been shown to terminate the AT. In this study, we extend this approach to scar-related ventricular tachycardia (VT), complicating the calculations as VT is inherently a 3-dimensional problem. We propose that scar-related VT can be topologically simplified to one or more of four basic physiologically distinct scar-types: transmural (I-shaped), epicardially connected or endocardially connected (U-shaped) or intramural (O-shaped).

**Methods:**

Six simulations of scar-related VT were created, each featuring a distinct critical scar configuration. From each simulation, three transmural layers (endocardium, mid-myocardium and epicardium) were extracted to create 2-dimensional surfaces, which were analyzed with the index theorem, using the software package Directed Graph Mapping (DGM) extended with novel algorithms to detect the CBs.

**Results:**

On each layer, either no CBs were found or pairs of counter-rotating CBs were found, each CB had an opposite sign, adhering to the index theorem. Ablation was performed by connecting each pair of counter-rotating CBs on each layer to form a continuous ablation surface, bounded by scar tissue, the endocardial surface, or the epicardial surface. This ablation strategy consistently terminated all simulations, supporting the applicability of our topology-based approach to VT.

**Conclusion:**

The index theorem remains valid for scar-related VT. Successful ablation on VT should include, connecting the CB-pairs in each 2 dimensional surface. Any other type of ablation does not terminate the VT.

## 1 Introduction

Ventricular tachycardia (VT), a common and potentially life-threatening arrhythmia, frequently arises from scar-related reentry in patients with structural heart disease. This abnormal electrical activity within the ventricles can compromise cardiac function, necessitating prompt intervention.

VT is believed to be driven either by triggered activity, where localized sources provide ectopic electrical activity, or by reentrant circuits, being continuous loops of electrical activity that are able to maintain itself through the constant circuitous re-excitation of tissue (Pogwizd et al., 1992; De Bakker et al., 1988). Conventionally, reentrant VT is believed to be sustained by either (i) a single reentry circuit, (ii) a dual-circuit pattern involving two reentry circuits with one active and one passive circuit, or (iii) by a dual-circuit pattern involving two active reentry circuits (Martin et al., 2018; Martin et al., 2019; Tung et al., 2020; Nishimura et al., 2021a; Hawson et al., 2022). In cases of dual-loop reentry with only one active circuit (ii), the passive loop is not considered to maintain the VT.

The standard therapeutic approach aiming to eliminate VT is called catheter ablation, which entails selectively ablating regions, effectively burning tissue, which in turn prevents the perpetuation of the electrical activity. In this context, precise targeting is crucial, as excessive ablation can negatively impact ventricular function and cause additional complications (Stevenson et al., 2008).

Following the identification of the mechanism, the most suitable ablation target is identified, known as the protected VT isthmus. Identifying this isthmus can be particularly challenging, especially in hemodynamically unstable 5T cases (Zeppenfeld et al., 2022). As a result, clinicians often aim to characterize the arrhythmogenic substrate by identifying scar tissue using cardiac magnetic resonance imaging (Roca-Luque et al., 2020) or computed tomography scans (Kuo et al., 2019), which can help in localizing the isthmus.

Additionally, many studies point out that 3D substrate-based research is required to increase the understanding of arrhythmia mechanisms and improve ablation outcome in this heterogeneous patient population, certainly for non-ischemic scar related VTs (Tung et al., 2020; Janneke et al., 2024; Campos et al., 2021; de Riv et al., 2018; Proietti et al., 2015). For this purpose, we use concepts of topology introduced in previous research (Nele et al., 2024; Duytschaever et al., 2024; Abeele et al., 2025). In this work, we apply the index theorem, which states that the sum of the topological indices around all boundaries on a closed surface must equal zero (Winfree and Tyson, 1988; Davidsen et al., 2004; Herlin and Vincent, 2012). Applied to reentrant atrial tachycardia (AT), this implies that reentrant loops always occur in opposing pairs: one clockwise (CW) loop with an index of −1 and one counter-clockwise (CCW) loop with an index of +1. Of note, near-complete reentrant circuits, often labeled as passive or bystander loops, also carry a nonzero index, making them equally significant to complete loops. As such, a boundary with a nonzero index was defined as a critical boundarys (CBs) (Santucci et al., 2023), whereas boundaries that exhibit an index of 0 were classified as non-critical boundary (NCB). In order to terminate the AT, the CBs must be connected by an ablation line.

Building on these successful results (Nele et al., 2024; Duytschaever et al., 2024; Abeele et al., 2025), we hypothesize that reentry loops during VT also occur as paired structures. Consequently, we propose that true single-loop reentry does not exist in VT. Therefore, the traditional distinction between “active” and “passive” loops, where only the active loop is thought to drive the arrhythmia, is likely incorrect.

However, since the ventricles cannot be seen as a 2D surface, layers of this ventricle would need to be extracted before applying the index theorem. As such, we hypothesize that the index theorem applies to each individual layer of the ventricular wall, meaning that the epicardium, endocardium, and any intermediate layers must each independently satisfy the index theorem. Accordingly, if a reentry loop is present in a given layer, there must also be a second, counter-rotating loop with an opposite index within that same layer. When reentry occurs, at least one of these loops will be complete, while the second may be complete or near-complete. Previously, such near-complete loops have been labeled as “passive”. However, we will demonstrate that these near-complete loops contribute equally to the maintenance of VT and should not be considered passive. Importantly, we will demonstrate that it is possible for some layers to exhibit two reentry loops, while other layers may show no reentry activity at all. However, even loop-free layers can exhibit focal activation patterns that are driven by reentry loops in adjacent layers. Only by integrating information across all layers can a complete three-dimensional representation of the CB be constructed. To successfully terminate the VT, these CBs must be interconnected within each layer to form a continuous surface that blocks electrical activity.

**FIGURE 1:**
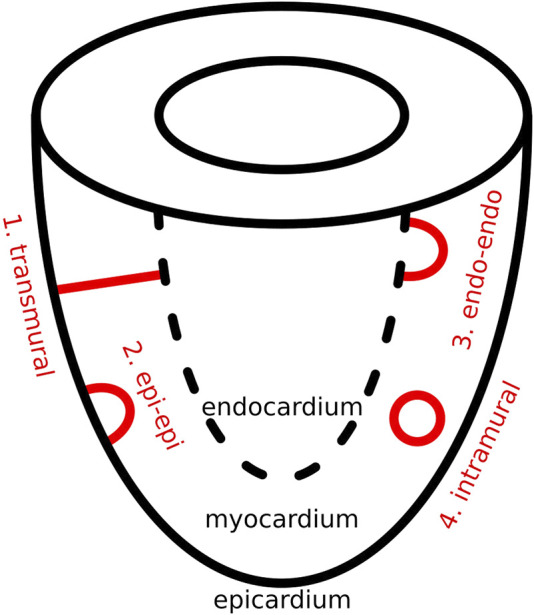
Overview of four physiologically distinct scar types during VT.

In this proof-of-concept study, we focus exclusively on scar-related VT. In this context, using topological deformation, we propose that each complex scar structure can be conceptually divided into two components: a non-critical part, which does not contribute to reentrant activity, and a critical part, which serves as the center for rotational activity. The critical part can be further simplified into one or more of three fundamental topological configurations: I-shaped, O-shaped, and U-shaped structures. From a physiological perspective, these three topological forms give rise to four distinct reentry patterns, as discussed below and as can be seen in Figure 1.

The same four structural types have previously been proposed in studies of ventricular fibrillation (VF) as the different possible shapes of filaments (Arthur and Strogatz, 1984; Clayton et al., 2006; Pertsov et al., 2000). In the present work, we reinterpret this concept by substituting filament geometry with the anatomical structure of dense scar tissue. When reentry is constrained by the geometry of the scar, the resulting center of rotation aligns with the filament core described in earlier models. In this way, a clear analogy emerges between the filament shapes and the structural patterns formed by the scar. Additionally, to introduce this concept an idealized substrate of a single ventricle was chosen.

## 2 Methods

### 2.1 Four basic scar configurations

**FIGURE 2:**
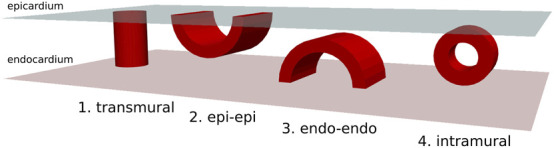
3-dimensional overview of four possible physiologically distinct scar types (Red). The epicardium is represented in light-blue and the endocardium in pink.

As previously mentioned, in scar-related VT, we reduced the critical scar to a combination of four basic physiological types. These four critical scar types are represented in Figure 1 and are further illustrated in 3D in Figure 2.

• (1) A transmural scar: This I-shaped scar connects the endocardium layer to the epicardial layer, creating a transmural scar. Of note, the thickness of this scar may vary, provided that reentry remains feasible and the end-points interconnect the epi- and endocardium.

• (2) An epi-epi or (3) an endo-endo scar: These types of scar are U-shaped and are connected end-to-end to the same myocardial wall, using an arch-shaped structure with a certain thickness and a conduction pathway through the arch. Epi-epi scars connect the epicardium to epicardium via the mid-myocardium and endo-endo scars connect the endocardium to endocardium via the mid-myocardium. Most notably, the dimensions of the arch-shaped scar may range from a thin arch to a more tube-like configuration, provided that reentry remains feasible and a conduction pathway underneath the arch is preserved.

• (4) An intramural scar: Finally, it is also possible to have an intramural scar, which is entirely embedded within the myocardium and thus completely disconnected from both the epi- and endocardium. This scar forms a ring within the ventricular wall by connecting to itself, giving rise to an O-shaped scar with a certain thickness, and an intramural pathway through this ring. It is important to note that the dimensions of this ring may vary, ranging from very thin structures to those that are more tube-like in configuration, provided that an intramural conduction pathway within the ring is present.

Using these scar-types, we performed six different types of simulations. The exact configuration for each simulation is described in Section 2.3.

### 2.2 Simulation parameters for VT induction

Simulations were performed with openCARP (Plank et al., 2021), an open cardiac electrophysiology simulator for *in silico* experiments. More specific parameters are discussed in following paragraphs.

Single cell model: For our cellular model, we used a ventricular cell model, namely, the Ten Tusscher-Panfilov model (TP06) (J ten Tusscher and Panfilov, 2006), which was modified to facilitate reentry by shortening the action potential duration. These modifications included reducing the L-type calcium conductance $G_{\mathrm{CaL}}$ by 70% and increasing the rapid delayed rectifier potassium conductance $G_{Kr}$ by 70%, the inward rectifier potassium conductance $G_{k1}$ by 80%, and the slow delayed rectifier conductance $G_{Ks}$ by 80%. To ensure steady-state conditions, this modified model was prepaced at a 250 m interval (S1-S1) for 50 s.

Mesh parameters: As a proof-of-concept for VT, we constructed an abstracted model of the left ventricle as follows: a tetrahedralized sphere with a 40 mm radius and a wall thickness of 20 mm was generated. To represent the ventricular base, the top 27.5 mm of the sphere was removed, yielding an idealized single-ventricle geometry. The spatial resolution of the mesh was 783 μ m. A relatively slow conduction velocity of 0.3 mm/ms (Aronis et al., 2020) was imposed to promote reentry induction, however, this reduced velocity does not affect the underlying topology of the resulting arrhythmia.

Reentry induction protocol: We used the following protocol to induce reentry in our model. First, the designated scar type (Figure 1) was added to the mesh. Then, five consecutive pulses with an interval of 250 m were applied in a specific location. During this final stimulus, a temporary block was introduced to one side of the pulse represented by a surface, bound entirely by either scar, the epi- and/or endocardium. This temporary block was then removed 200 m after the induction on the last stimulus, thereby inducing reentry. Finally, the simulation was allowed to stabilize for 1800 m.

### 2.3 Simulations

Using the protocol from the previous section, in total six different scenarios were created, each with a different scar configuration or induction location.

**FIGURE 3:**
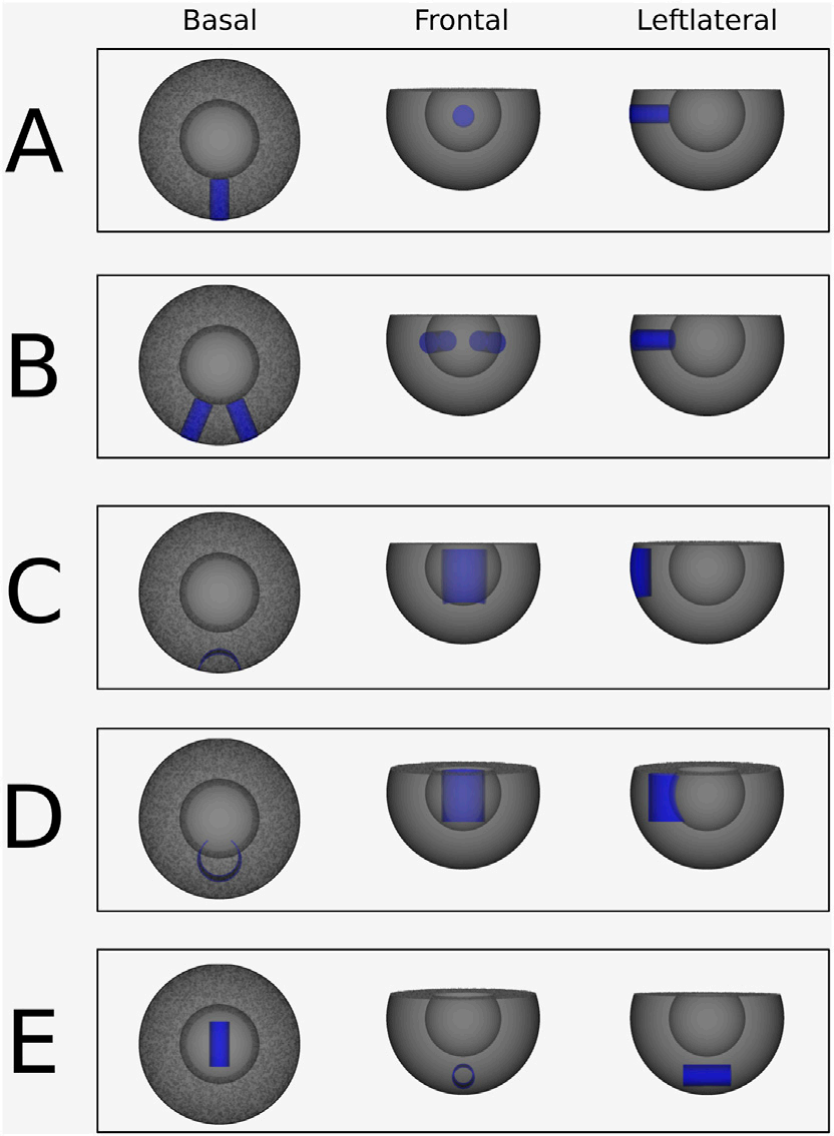
Abstracted ventricular substrates used for the simulations. Healthy myocardium is shown in grey, and non-conductive scar tissue is highlighted in blue. **(A)** Substrate, used for the simulation 1. **(B)** Substrate, used for the simulation 2 and 3. **(C)** Substrate, used for the simulation 4. **(D)** Substrate, used for the simulation 5. **(E)** Substrate, used for the simulation 6.

Simulation 1: A single transmural scar. In this simulation, a cylindrical scar with a radius of 12 mm was embedded in the substrate, extending continuously from the epicardium to the endocardium through the myocardial wall (Figure 3A). To induce reentry, series of pacing pulses was applied between the scar and the ventricular base. Just before the final pulse, a temporary conduction block was introduced on one side of the stimulus pathway between the scar and the base. This block was removed 200 m later, enabling the initiation of reentry around the scar.

Simulation 2: Two transmural scars. A VT was simulated with two separate transmural scar structures. Both scars are cylinders with a radius of 12 mm and lie in the xy-plane and at and angle of 45° to each other (Figure 3B). Here, the pulse was placed in between both scars, with the temporary block right above the pulse connecting the two scars, preventing upwards conduction.

Simulation 3: Two transmural scars with different VT Induction. This transmural scar simulation was performed using the same configuration as simulation 2 (Figure 3B), but with a different pacing site. In this case, the pulses were applied between the ventricular base and the left transmural scar, accompanied by a temporary conduction block positioned to the left of the stimulus.

Simulation 4: Epi-epi connected scar. An epi-epi scar was introduced by removing an open cylindrical segment from the mesh. The cylinder measured 25 mm in height and 20 mm in radius, with its geometric center located at (20 mm, 0 mm, −2.5 mm). This configuration ensured that the cylinder intersected the mesh surface exclusively at the epicardium, as shown in Figure 3C. All points on the cylinder’s surface were designated as non-conductive, creating an arch-shaped scar that originates and terminates at the epicardium while traversing the mid-myocardium without reaching the endocardium. Reentry was induced according to the standard protocol, with pacing pulses delivered within the cylinder and a temporary conduction block applied at the top of the cylinder.

Simulation 5: Endo-endo connected scar. Similar to the epi-epi scar, the endo-endo scar was created by removing an open cylindrical segment from the mesh. The cylinder had a height of 25 mm and a radius of 20 mm, with its geometric center positioned at (40 mm, 0 mm, −2.5 mm). This configuration ensured that the cylinder’s surface intersected the mesh only at the endocardium, as illustrated in Figure 3D. Points on the cylindrical surface were designated as non-conductive, resulting in an arch-shaped scar that originates and terminates at the endocardium, passing through the mid-myocardium without reaching the epicardium. Reentry was induced following the standard protocol, with pacing pulses delivered within the cylinder and a temporary conduction block applied at its top.

Simulation 6: Intramural O-shaped scar. The intramural scar was introduced as an open cylindrical structure with a radius of 5 mm, a length of 25 mm, and a geometric center at (0 mm, 0 mm, −30 mm), embedded horizontally within the myocardial tissue (Figure 3E). This configuration resulted in a closed, ring-like scar that did not intersect either the epicardial or endocardial surfaces, and was therefore classified as an intramural scar (Figure 1, panel 4). Reentry was induced according to the established protocol, with pacing pulses applied within the cylinder and a temporary conduction block placed at one end.

### 2.4 Topological charge calculation

In this section, we describe the method used to compute the index, or topological charge (TC), of a given boundary, as applied in the simulations discussed in the previous section.

The TC of a region A is defined by Equation 1 as the closed path integral of the spatial phase gradient along its boundary C , traversed CCW, and normalized by 2π . Of note, this quantity is well-defined if the surface A is a two-dimensional orientable compact connected manifold with a finite number of holes, and the phase field ϕ(x) is defined in A as a real function with values modulo 2 π , which is smooth everywhere in A except at a finite number of points (Davidsen et al., 2004; Herlin and Vincent, 2012).

$$\text{TC}=\frac{1}{2\pi }\oint_{C}\vec{\nabla }\phi \cdot \vec{d\ell }$$ (1)

In Equation 1, $\vec{\nabla }\phi$ represents the spatial gradient of the phase and $\vec{d\ell }$ the tangent to the C . As a tachycardia is a periodic arrhythmia, we interpret this normalized phase ϕ/2π as the local activation time (LAT), normalized by the tachycardia cycle length (TCL). As such, we can redefine Equation 1 as:

$$\text{TC}=\frac{1}{\text{TCL}}\oint_{C}\vec{\nabla }\text{LAT}\cdot \vec{d\ell }$$ (2)

Using Equation 2, the TC can be computed for each anatomical boundary. A boundary with a non-zero TC is classified as a CB, whereas a boundary with a TC of zero is labeled as a NCB. In previous work, we developed an algorithm to compute the TC around boundaries in mapped cases of AT.

To perform these calculations, we used the DGM-TOP algorithm (Abeele et al., 2025), which is part of our software tool called Directed Graph Mapping (DGM), a framework that applies network theory to the analysis of cardiac arrhythmias. In this algorithm, all points along the identified boundaries are extracted, and Equation 2 is applied, allowing for the automatic detection of CB-pairs. This approach was validated in the context of AT, using over 500 AT simulations and 30 clinical AT cases (Abeele et al., 2025). In the present work, we applied the same algorithm to our simulations. However, while in AT there is only a single surface to consider, in VT we extended this approach by applying the algorithm to three extracted layers of the ventricular wall: the epicardium, a mid-ventricular layer, and the endocardium.

More specifically, the DGM-TOP algorithm performed the following steps. First, after stabilization of the simulation, all points were annotated with an LAT-value by identifying the moment in time at which the transmembrane voltage exceeded −40 mV, corresponding to the passing of the depolarization wave.

Second, 2D surfaces were extracted from the 3D substrate by selecting all vertices and cells located at specific radial distances from the center of the spherical mesh. This process yielded three distinct layers: the endocardium, mid-myocardium, and epicardium, corresponding to radii of 22 mm, 30 mm, and 38 mm, respectively.

**FIGURE 4:**
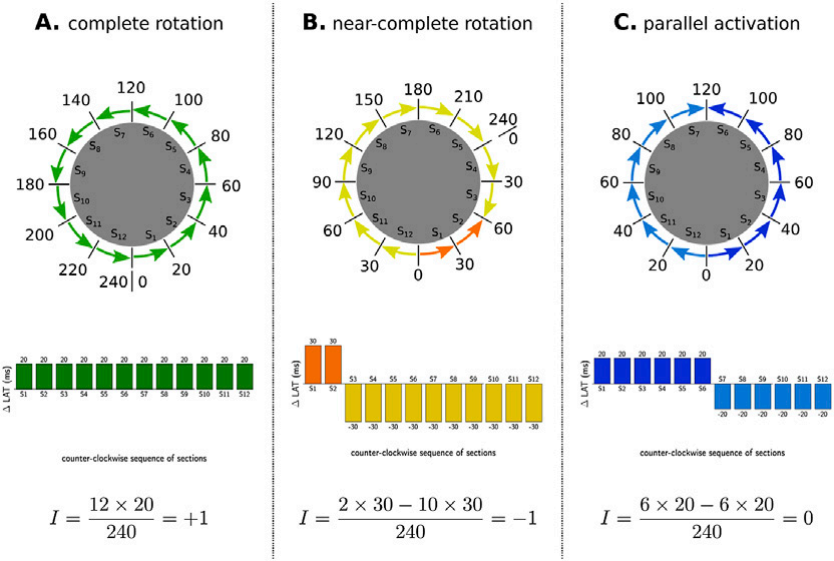
Examples of index calculation on three observed patterns in a tachycardia with TCL of 240 m. **(A)** represents a CCW reentry, as such al ΔLAT are positive, and sum up to 240 m, creating a TC of +1. **(B)** shows a near-complete rotation and has a large CW part, of which the ΔLAT sum up to −300 m and a small CCW part that sums up to +60 m, creating a TC of −1. **(C)** depicts parallel activation with a CCW part of −120 m and a CW part of 120 m, creating a TC of 0. Through this index calculation, **(A,B)** are considered CB, while **(C)** is considered an NCB.

Subsequently, all scar nodes were removed, and the boundary points enclosing each remaining region were sequentially identified. Since the mesh is inherently a discretized structure, the continuous integral (Equation 2) for computing the TC was re-defined as a Riemann sum over all boundary points ( $S_{i}$ ), sequentially traversed in the CCW direction. In this discretized form, the gradient of LAT in the integral is replaced by the finite difference $\Delta {\text{LAT}}_{i}$ (Equation 4) between consecutive neighboring boundary segments ( $S_{i}$ ), resulting in Equation 3. As such, boundaries with either a complete or near-complete rotation exhibit a non-zero TC and are labeled as CBs, while boundaries without rotational activity exhibit a TC of 0 and are labeled NCBs. Figure 4 shows examples of these three types of pattern.

$$\text{TC}=\frac{1}{\text{TCL}}\underset{S_{i}}{\sum }\Delta {\text{LAT}}_{i}$$ (3)

$$\Delta {\text{LAT}}_{i}={\text{LAT}}_{i+1}-{\text{LAT}}_{i}$$ (4)

**FIGURE 5:**
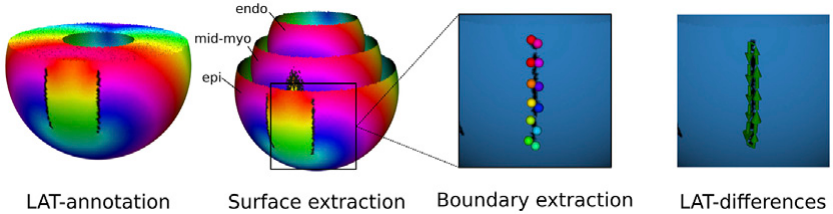
DGM-TOP pipeline for ventricular tachycardia. First LATs were annotated. Second, three surfaces (endocardium, mid-myocardium and epicardium) were extracted. Third, the boundaries were located and their points extracted. Fourth, the TC was calculated for each boundary.

An example of entire pipeline is shown in Figure 5.

### 2.5 Ablation protocol

Following the identification of the CBs, ablation lines were placed to interconnect the CBs of opposite signs within each layer. Together, these lines form a continuous surface, bounded by the scar tissue, the epicardial surface and/or endocardial surface. For detailed placement of these ablation lines, we refer to the Results section (Section 3).

## 3 Results

### 3.1 Transmural scar

**FIGURE 6:**
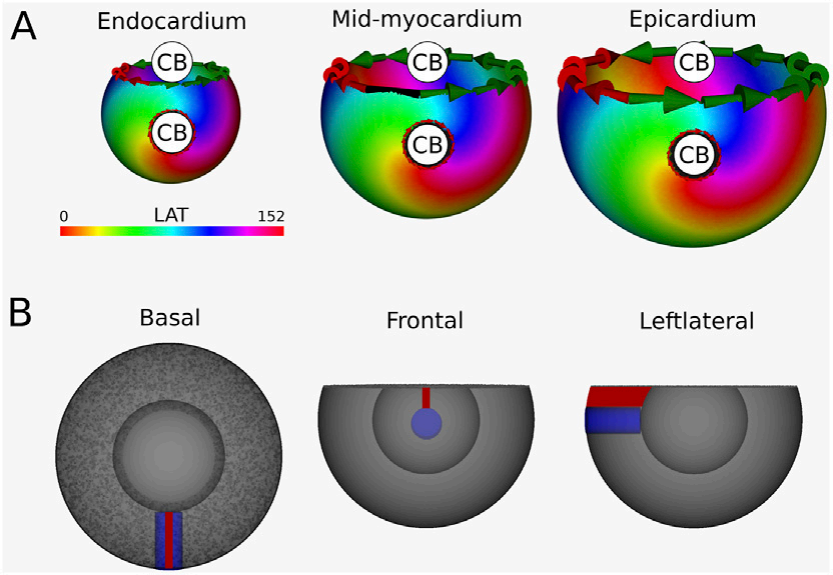
**(A)** LAT-map of the first simulation with transmural scar. 2 CBs are found in each of the three ventricular layers: one around the transmural scar and one around the base of the ventricle. These critical boundaries are indicated by a label and accompanying arrows around its boundary: Red for CCW and Green for CW conduction. **(B)** Ablation lesion (red) that terminated the simulation.

Simulation 1: A single transmural scar. Induction of reentry around a single transmural scar resulted in the creation of a VT with a TCL of 152 m. The transmembrane voltages of the simulation can be viewed in Supplementary Video 1 and in Figure 6 the LAT-map of the simulation is shown.

Computation of the TC in endocardium, mid-myocardium and epicardium confirmed a reentrant circuit (complete rotaion) with TC=−1 around the induced scar, in addition to a second CB (near-complete rotation) with TC=+1 around the base of the ventricle as calculated by Equation 3. This finding was consistently observed across all three layers.

Per protocol, an ablation lesion was created, connecting the transmural scar with the base of the ventricle in each 2D layer, in such a way as to form a continuous surface of non-conductive tissue, thereby terminating the VT.

**FIGURE 7:**
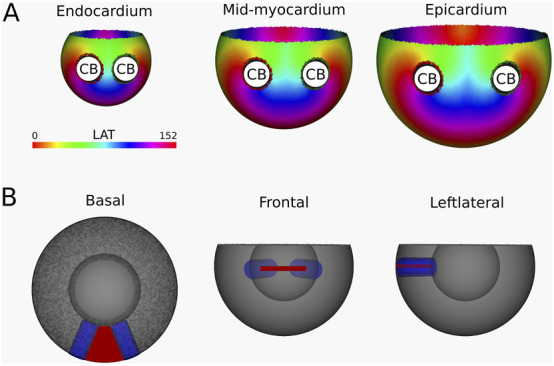
**(A)** LAT-map of the first simulation with two transmural scars. 2 CBs are found in each layer: one around each transmural scar. The base exhibited a TC of 0, classifying it as a NCB. The critical boundaries are indicated by a label and accompanying arrows around its boundary: Red for CCW and Green for CW conduction. **(B)** Ablation lesion (red) that terminated the simulation.

Simulation 2: Two transmural scars. This simulation resulted in a VT with cycle length 152 m. Calculations of the TC revealed a CB-pair in each transmural layer. This pair consists of the boundaries of the two transmural scars, around which two reentry circuits (complete rotations) are located, as illustrated in the LAT-map (Figure 7) and Supplementary Video 2. The left scar exhibits a TC of −1, while the right scar shows a TC of +1.

Following identification of the CBs, an ablation lesion was imposed on the tissue, connecting the two transmural scars in each transmural layer, in the shape of a continuous surface of non-conductive tissue. This technique terminated the simulated VT.

**FIGURE 8:**
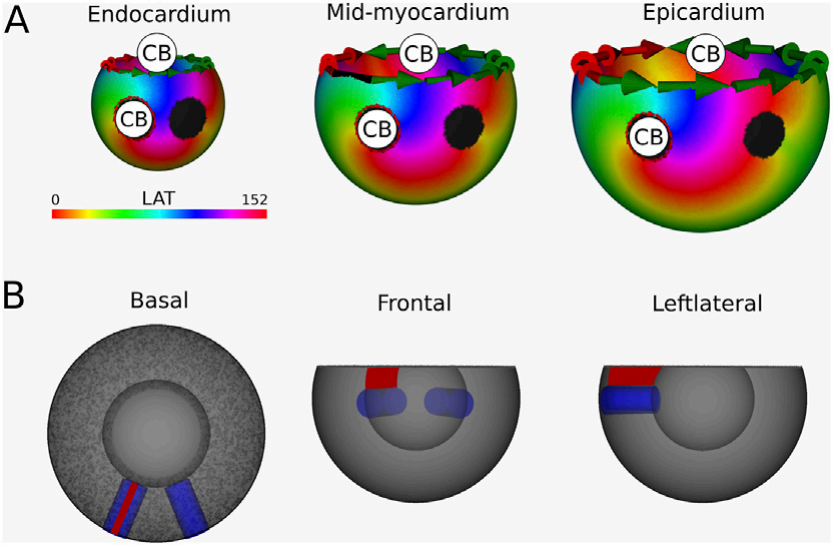
**(A)** LAT-map of the second simulation with two transmural scars. 2 CBs are found in each layer: one around the left transmural scar and the other around the base. The leftmost scar exhibited a TC of 0, labeling it as a NCB. The critical boundaries are indicated by a label and accompanying arrows around its boundary: Red for CCW and Green for CW conduction. **(B)** Ablation lesion (red) that terminated the simulation.

Simulation 3: Two transmural Scars with Different VT Induction. This simulation utilizes the same substrate as Simulation 2, albeit with a different induction location. As such, a VT with a cycle length of 152 m was created, where a CB (complete rotation) with TC = +1 was consistently found around the left transmural scar and another CB (near-complete rotation) with a TC of −1 around the base of the ventricle. The LAT-map of this simulation is shown in Figure 8, with the simulation in Supplementary Video 3.

After identification of the CBs, an ablation lesion was created similarly as in Simulation 1, interconnecting the left most transmural scar to the base of the ventricle in each layer, terminating the VT.

**FIGURE 9:**
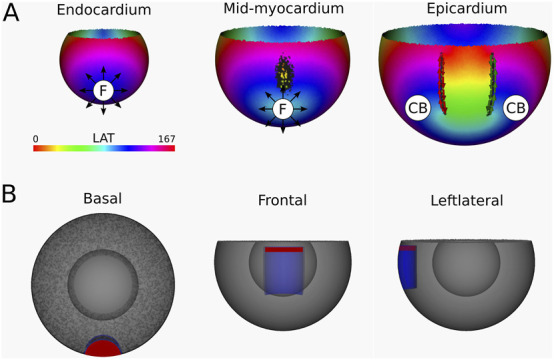
**(A)** LAT-map of the simulation of reentrant VT with circuits around the epi-epi scar (Figure 1 (2)). 2 CBs are found in the epicardial layers. The left CB has a TC of −1, while the right CB has a TC of +1. No CBs are found in the endocardial layers. The base of the model exhibited a TC of 0, thereby classifying it as a NCB. The critical boundaries are indicated by a label and accompanying arrows around its boundary: Red for CCW and Green for CW conduction. Additionally, focal activity is indicated by F. **(B)** Ablation lesion (red) that terminated the simulation.

Simulation 4: Epi-epi connected scar. Following the reentry induction protocol, Figure 9 shows a VT with a TCL of 167 m. Starting from the epicardial layer, DGM-TOP identifies two CBs with opposite TCs located at the ends of the scar. Both CBs are complete rotations. In the mid-myocardium the two boundaries converge, and the CBs disappear. As such, in the mid-myocardium and the endocardium, no CBs are detected. However, focal activation patterns emerge, driven by reentrant waves originating from the epicardium, as illustrated in Supplementary Video 4. Importantly, in all layers, the total TCs remains zero, in accordance with the index theorem.

From the epicardium to the mid-myocardium, the identified CB-pair was interconnected via ablation. These lesions formed a continuous non-conductive surface that effectively blocked conduction around the arching epi-epi scar. As a result, reentry beneath the arch was prevented, successfully terminating the VT.

After applying the ablation lesion, the focal sources at the endocardium and the mid-myocardium terminated, confirming that these sources were not the driving mechanism, but merely a epiphenomenon, caused by the reentry circuit in one of the other layers.

**FIGURE 10:**
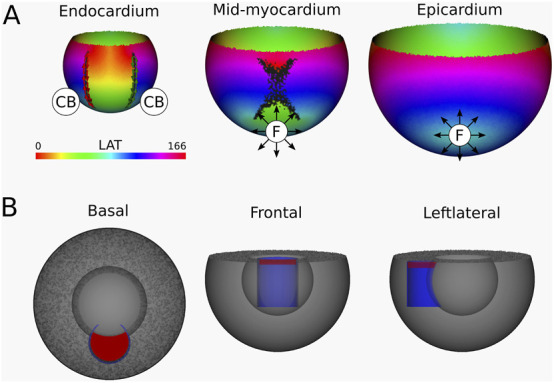
**(A)** LAT-map of the Simulation of VT with reentry around an endo-endo scar (Figure 1 (3)). In the endocardial layers, 2 CBs are found. The left CB and the right CB has a TC of −1. The base of the model exhibited a TC of 0, thereby classifying it as a NCB. The critical boundaries are indicated by a label and accompanying arrows around its boundary: Red for CCW and Green for CW conduction. Additionally, focal activity is indicated by F. **(B)** Ablation lesion (red) that terminated the simulation.

Simulation 5: Endo-endo U-shaped connected scar. The resulting simulation yields a VT with a TCL of 166 m, visualized in Figure 10 and shown in Supplementary Video 5. Starting from the endocardial layer, DGM-TOP identifies two CBs with opposite TCs located at the ends of the scar, both are complete rotations. In the mid-myocardium, the two boundaries converge, and no CBs are detected. Finally, on the epicardium, no CBs are detected. Instead, focal activation patterns are observed, resulting from reentrant activity propagating from the endocardium. Despite these changes, the total TCs remains zero in all layers, in accordance with the index theorem. Of note, Simulations four and five can be regarded as mirror images of each other, both representing U-shaped scars.

From the endocardium to the mid-myocardium, the identified CB-pair was interconnected via ablation. These lesions formed a continuous non-conductive surface that effectively blocked conduction around the arching endo-endo scar. As a result, reentry beneath the arch was prevented, successfully terminating the VT.

Similarly to Simulation 4, after applying the ablation lesion, the focal sources at the epicardium and the mid-myocardium terminated, confirming that these sources were not the driving mechanism, but merely a epiphenomenon, caused by the reentry circuit in one of the other layers.

**FIGURE 11:**
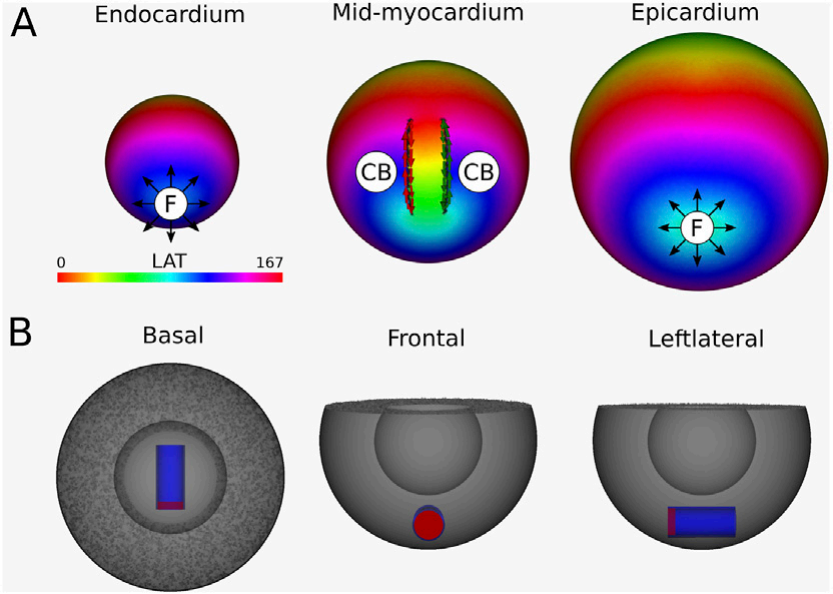
**(A)** LAT-map of the VT-simulation of intramural O-shaped scar (Figure 1 (4)). Only in the mid-myocardial layer, 2 CBs are found. The left CB has a TC of −1, while the right CB has a TC of +1. The base of the model exhibited a TC of 0, thereby classifying it as a NCB. The critical boundaries are indicated by a label and accompanying arrows around its boundary: Red for CCW and Green for CW conduction. Additionally, focal activity is indicated by F. **(B)** Ablation lesion (red) that terminated the simulation.

Simulation 6: Intramural O-shaped scar. In this simulation, a VT with TCL of 167 m was observed. The simulation is shown in Supplementary Video 6, with the corresponding LAT map presented in Figure 11. DGM-TOP identifies CB-pairs exclusively in the mid-myocardial layers, which correspond to the region fully traversed by the intramural cylinder. Both CBs inhibit complete rotations. No CBs are detected in layers toward the epicardium or endocardium. In these outer layers, focal activation patterns are observed, driven by the intramural reentry. Across all layers, the total TCs remains zero, in agreement with the index theorem.

To stop the VT, in all myocardial layers where a CB-pair was found, the pair was interconnected by non-conductive tissue. This ablated tissue formed a circle-like surface, blocking the entrance of the O-shaped scar, thereby preventing conduction inside the scar and terminating the simulation.

Similarly to Simulation 4 and 5, after applying the ablation lesion, the focal sources at the endocardium and the epicardium terminated, confirming that these sources were not the driving mechanism, but merely a epiphenomenon, caused by the reentry circuit in one of the other layers.

## 4 Discussion

In this manuscript, we explored the application of the index theorem to an abstracted ventricular shape with various simplified scar configurations. By extracting multiple 2D layers from the 3D shape, we were able to apply TC calculations to locate the CB. In each of the basic cases examined, the DGM-TOP algorithm consistently identified two CBs forming a pair with opposite rotation, as indicated by green and red arrows. This finding demonstrates that each positive TC is balanced by a negative TC, resulting in a total TC of zero for each separate layer, thus preserving the integrity of the index theorem.

The fundamental concept of ablation in VT remains analogous to ablation in AT: pairs of counter-rotating CBs must be connected to terminate the tachycardia. However, a crucial distinction in VT ablation is the necessity to consistently connect the CB-pair across all extracted layers, effectively creating a surface of ablated tissue. This three-dimensional approach reflects the complex nature of ventricular arrhythmias and underscores the importance of comprehensive mapping of both epicardial and endocardial layers. A focal trigger observed on the epicardium or endocardium may still indicate the presence of reentry in the opposite layer (Simulations 4–5) (Jeffrey et al., 1985). Moreover, simultaneous focal triggers on both the epicardium and endocardium can be the surface manifestation of an intramural reentry within the myocardial wall (Simulation 6) (Pogwizd et al., 1992).

Finally, if more complex critical scar-structures can be reduced to combinations of the proposed basic scar types (intramural, epi-epi, endo-endo, or transmural), then this framework could substantially simplify the characterization of VT and reduce ambiguity in the analysis of clinical cases. However, further clinically accurate data are required to validate this hypothesis.

### 4.1 Comparison of existing topological approaches with our work

Previous work has explored the role of topology in ventricular arrhythmia. Most recently, Arno et al. (Arno et al., 2024) proposed a topological framework for dynamic phase defects in excitable media, introducing structures such as head, tail, and pivot curves to describe the evolving wave patterns characteristic of fibrillation. In contrast, our work focuses on ventricular tachycardia characterized by critical scar, which can be described as stationary phase defects.

More fundamentally, Clayton et al. (Clayton et al., 2006) investigated possible rotor-filament structures in three-dimensional tissue, proposing I-, U-, and O-type structures, which correspond to the scar morphologies we present in Section 1. Their observation that U- and O-type structures can terminate by moving to the boundary or contracting aligns with these findings, as these processes effectively link clockwise and counter-clockwise rotations, leading to pair annihilation.

However, a key difference between this work and that of Clayton et al. (Clayton et al., 2006) lies in the treatment of the boundary. While we studied the arrhythmia in a more conceptually accurate ventricular shape, the authors in Clayton et al. used a tissue slab. Therefore, the contribution of the boundary was overlooked. Deforming the slab into a ventricular shape reveals that the boundary of the slab effectively acts as the inverse of the ventricular base. Consequently, including the TC of this boundary results in a total conserved TC of zero. Additionally, Clayton et al. handles dynamic rotation centers (rotor-filaments), while this manuscript static rotation centers (critical scar).

### 4.2 Potential clinical implications

Catheter ablation is a standard clinical approach to VT elimination, as discussed in the introduction. In general, electrophysiological studies show three different mechanisms of VT. As stated in the introduction, VT are considered to be driven by different reentry configurations: (i) a single reentry loop, (ii) dual reentry loops with one active and one passive component, with the active component driving the TCL or (iii) dual active reentry loops, also known as F8 reentry (Martin et al., 2018; Martin et al., 2019; Tung et al., 2020; Nishimura et al., 2021a; Hawson et al., 2022).

However, our topological analysis reveals that isolated single reentry loops do not occur. Instead, all reentry-driven VTs must satisfy the index theorem. This implies that within each layer, such as the epicardium or endocardium, a reentry loop with positive TC (counterclockwise rotation) must be accompanied by a loop with negative TC (clockwise rotation). An *active* loop corresponds to a complete reentrant circuit, while a *passive* loop is an near-complete loop as was show in prior research (Duytschaever et al., 2024). Nevertheless, both exhibit a TC of ±1 and contribute equally to sustaining the VT.

Moreover, effective ablation strategies should aim to interrupt the connection between the core structures of both rotations. These cores should be considered as three-dimensional structures, as illustrated in our six main simulations. Therefore, an ablation strategy should be considered, which connects CBs in such a way as to create a continuous surface, blocking conductivity between the CBs throughout the entire transmural depth of the ventricle. This ablation strategy appears to coincide with the ablation of the protected VT isthmus. However, this study is a pure conceptual study. Validation with clinical data is required to confirm the hypotheses stated in this paper.

### 4.3 Limitations

The bowl model used in this paper is limited as it does not include the distinction of the two anatomical holes in each ventricle (AV + MV or TV + PV). Additionally, it visually distinguishes the base area from the epicardium with a sharp edge, while realistically there is no such distinction.

The cycle lengths observed in our simulations are significantly shorter than those typically seen in clinical VT cases (Stevenson et al., 1997; Nishimura et al., 2021b). This is mainly because we did not incorporate slow-conducting zones, which can greatly contribute to the cycle length (Martin et al., 2018; De Bakker et al., 1988) or MRI-based ionic remodeling. Our goal was to maintain a simplified substrate in order to clearly illustrate the core topological concepts. While introducing realistic ventricular anatomy, heterogeneous conduction, and patient-specific remodeling would bring the simulations closer to clinical scenarios, we deliberately opted for a conceptual model rather than aiming to construct digital twins of specific VTs.

Due to the homogeneous nature of the tissue and the symmetry of certain scar-structures, only two of the six simulations contained near-complete rotations. However, as discussed in (Duytschaever et al., 2024), these near-complete rotations behave similar to the complete rotations, counting towards the total TC of the surface. Therefore, adding this type of rotation in these proof-of-concept cases would add little value.

we restricted our analysis to three ventricular layers (epicardium, mid-myocardium, and endocardium). In principle, however, the index theorem applies to any continuous surface within the ventricles (excluding the base). Since one layer can be smoothly deformed into another, the results should hold across all layers. Nonetheless, future work could explicitly test additional intermediate layers to further confirm the robustness of our findings.

The layer-based extraction discussed in this manuscript works well for this specific substrate. However, for more realistic ventricle models and more complex scar-shapes, a more sophisticated approach will be needed as the path of a reentry could be distributed over multiple different transmural layers.

In this manuscript, only scar-based VT was discussed, meaning that only anatomical reentry was taken into account. Discussing VT in the absence of scar requires the addition of functional reentry. However, functional reentries should not violate the index theorem as already discussed in previous literature concerning the creation and annihilation of pairs of counter-rotating rotors (Arno et al., 2023). As such, by implementing similar methods to identify functional block, this method could be extended to include this type of reentry as well.

The absence of mid-myocardial mapping in clinical settings makes it hard to detect and ablate O-type critical scar. However, the insights in our work may prove valuable if the technology advances to that point.

## 5 Conclusion

This manuscript proposes a simplification of the critical scar into a combination of base scar types. As such, calculation of TC reveals adherence to the index theorem on each two-dimensional extracted manifold of the three-dimensional. Termination of these simplified cases requires ablation by interconnecting CBs with opposite signs in such a way as to create a continuous surface.

## Data Availability

The raw data supporting the conclusions of this article will be made available by the authors, without undue reservation.

## Abbreviations

AT, atrial tachycardia; TC, topological charge; CCW, counter-clockwise; CW, clockwise; TCL, tachycardia cycle length; LAT, local activation time; CB, critical boundary; NCB, non-critical boundary; VT, ventricular tachycardia.
